# Supplementary material for: Multi‐dataset identification of innovative feature genes and molecular mechanisms in keratoconus
Source: J Cell Mol Med. 2024 Sep 19;28(18):e70079. doi: 10.1111/jcmm.70079 (PMC11412914; doi:10.1111/jcmm.70079)
Supplement: Supplementary file 2 — Material S2. [file JCMM-28-e70079-s002.zip › 7.GSEA analysis/2.ATOH7/KEGG_PROSTATE_CANCER.html]

Details for gene set KEGG\_PROSTATE\_CANCER[GSEA]

|  || Dataset | ATOH7.group.cls#h\_versus\_l |
| Phenotype | group.cls#h\_versus\_l |
| Upregulated in class | h |
| GeneSet | KEGG\_PROSTATE\_CANCER |
| Enrichment Score (ES) | 0.38356015 |
| Normalized Enrichment Score (NES) | 1.582933 |
| Nominal p-value | 0.013565891 |
| FDR q-value | 0.31411704 |
| FWER p-Value | 0.637 |
Table: GSEA Results Summary

  

Fig 1: Enrichment plot: KEGG\_PROSTATE\_CANCER      
 Profile of the Running ES Score & Positions of GeneSet Members on the Rank Ordered List

  

| SYMBOL | TITLE | RANK IN GENE LIST | RANK METRIC SCORE | RUNNING ES | CORE ENRICHMENT || 1 | AKT3 | na | 9 | 1.043 | 0.0852 | Yes |
| 2 | PDGFD | na | 133 | 0.739 | 0.1422 | Yes |
| 3 | PDGFC | na | 274 | 0.635 | 0.1902 | Yes |
| 4 | NKX3-1 | na | 301 | 0.624 | 0.2406 | Yes |
| 5 | CREB5 | na | 418 | 0.573 | 0.2842 | Yes |
| 6 | FGFR1 | na | 573 | 0.527 | 0.3229 | Yes |
| 7 | LEF1 | na | 735 | 0.492 | 0.3586 | Yes |
| 8 | BCL2 | na | 1098 | 0.432 | 0.3836 | Yes |
| 9 | TCF7 | na | 3059 | 0.277 | 0.3497 | No |
| 10 | PDGFB | na | 3642 | 0.250 | 0.3534 | No |
| 11 | MAP2K1 | na | 4240 | 0.228 | 0.3548 | No |
| 12 | CDKN1A | na | 5247 | 0.193 | 0.3416 | No |
| 13 | PIK3R3 | na | 6166 | 0.166 | 0.3287 | No |
| 14 | RELA | na | 6248 | 0.164 | 0.3397 | No |
| 15 | TCF7L1 | na | 6644 | 0.153 | 0.3409 | No |
| 16 | NRAS | na | 7693 | 0.126 | 0.3209 | No |
| 17 | NFKB1 | na | 8680 | 0.105 | 0.3010 | No |
| 18 | FGFR2 | na | 8886 | 0.101 | 0.3034 | No |
| 19 | INSRR | na | 9731 | 0.086 | 0.2861 | No |
| 20 | CREB1 | na | 9769 | 0.085 | 0.2920 | No |
| 21 | TGFA | na | 9931 | 0.083 | 0.2941 | No |
| 22 | EP300 | na | 10138 | 0.079 | 0.2946 | No |
| 23 | ARAF | na | 10150 | 0.079 | 0.3007 | No |
| 24 | HSP90B1 | na | 10223 | 0.078 | 0.3050 | No |
| 25 | GRB2 | na | 10441 | 0.075 | 0.3049 | No |
| 26 | NFKBIA | na | 10503 | 0.073 | 0.3092 | No |
| 27 | INS | na | 11499 | 0.059 | 0.2853 | No |
| 28 | CREB3L2 | na | 11898 | 0.054 | 0.2782 | No |
| 29 | CREB3L4 | na | 12000 | 0.053 | 0.2796 | No |
| 30 | EGFR | na | 12485 | 0.046 | 0.2694 | No |
| 31 | CDKN1B | na | 12681 | 0.044 | 0.2674 | No |
| 32 | EGF | na | 13899 | 0.031 | 0.2348 | No |
| 33 | CASP9 | na | 14196 | 0.029 | 0.2286 | No |
| 34 | HSP90AB1 | na | 14217 | 0.028 | 0.2304 | No |
| 35 | MAPK1 | na | 14536 | 0.025 | 0.2233 | No |
| 36 | HRAS | na | 14559 | 0.025 | 0.2247 | No |
| 37 | TP53 | na | 14698 | 0.024 | 0.2226 | No |
| 38 | RAF1 | na | 15219 | 0.019 | 0.2092 | No |
| 39 | IKBKG | na | 15619 | 0.016 | 0.1990 | No |
| 40 | CREB3 | na | 15750 | 0.015 | 0.1964 | No |
| 41 | AKT1 | na | 15787 | 0.014 | 0.1966 | No |
| 42 | GSK3B | na | 16028 | 0.013 | 0.1907 | No |
| 43 | PIK3CD | na | 16202 | 0.011 | 0.1866 | No |
| 44 | GSTP1 | na | 16750 | 0.007 | 0.1714 | No |
| 45 | MAPK3 | na | 16821 | 0.006 | 0.1699 | No |
| 46 | CREB3L3 | na | 16998 | 0.005 | 0.1652 | No |
| 47 | BAD | na | 17218 | 0.002 | 0.1590 | No |
| 48 | ATF4 | na | 17294 | 0.002 | 0.1570 | No |
| 49 | E2F3 | na | 17541 | -0.000 | 0.1499 | No |
| 50 | CDK2 | na | 18103 | -0.005 | 0.1341 | No |
| 51 | CTNNB1 | na | 18214 | -0.006 | 0.1314 | No |
| 52 | E2F1 | na | 18613 | -0.010 | 0.1207 | No |
| 53 | CREB3L1 | na | 19199 | -0.015 | 0.1050 | No |
| 54 | SRD5A2 | na | 19973 | -0.022 | 0.0845 | No |
| 55 | FOXO1 | na | 19979 | -0.022 | 0.0861 | No |
| 56 | CHUK | na | 20245 | -0.024 | 0.0805 | No |
| 57 | CCND1 | na | 20546 | -0.027 | 0.0740 | No |
| 58 | MAP2K2 | na | 20653 | -0.028 | 0.0733 | No |
| 59 | PIK3CB | na | 20685 | -0.028 | 0.0747 | No |
| 60 | HSP90AA1 | na | 21322 | -0.035 | 0.0592 | No |
| 61 | PIK3CA | na | 21359 | -0.036 | 0.0611 | No |
| 62 | TCF7L2 | na | 22134 | -0.044 | 0.0424 | No |
| 63 | PIK3R2 | na | 22209 | -0.045 | 0.0440 | No |
| 64 | PDGFA | na | 22257 | -0.046 | 0.0464 | No |
| 65 | SOS2 | na | 22550 | -0.049 | 0.0419 | No |
| 66 | RB1 | na | 22702 | -0.051 | 0.0417 | No |
| 67 | CCNE1 | na | 23221 | -0.058 | 0.0315 | No |
| 68 | IKBKB | na | 23356 | -0.060 | 0.0326 | No |
| 69 | PTEN | na | 23399 | -0.060 | 0.0363 | No |
| 70 | ERBB2 | na | 24092 | -0.071 | 0.0221 | No |
| 71 | MDM2 | na | 24258 | -0.073 | 0.0234 | No |
| 72 | KLK3 | na | 24301 | -0.074 | 0.0282 | No |
| 73 | PDPK1 | na | 24308 | -0.074 | 0.0341 | No |
| 74 | CREBBP | na | 24831 | -0.083 | 0.0258 | No |
| 75 | AR | na | 25031 | -0.086 | 0.0271 | No |
| 76 | BRAF | na | 25048 | -0.086 | 0.0337 | No |
| 77 | KRAS | na | 25083 | -0.087 | 0.0398 | No |
| 78 | MTOR | na | 25211 | -0.089 | 0.0435 | No |
| 79 | PIK3CG | na | 26210 | -0.109 | 0.0236 | No |
| 80 | SOS1 | na | 26852 | -0.122 | 0.0151 | No |
| 81 | AKT2 | na | 27111 | -0.128 | 0.0181 | No |
| 82 | IGF1R | na | 27632 | -0.142 | 0.0147 | No |
| 83 | PIK3R1 | na | 27720 | -0.144 | 0.0239 | No |
| 84 | CCNE2 | na | 28205 | -0.157 | 0.0228 | No |
| 85 | E2F2 | na | 30000 | -0.211 | -0.0117 | No |
| 86 | PDGFRB | na | 32781 | -0.349 | -0.0634 | No |
| 87 | IGF1 | na | 33039 | -0.370 | -0.0405 | No |
| 88 | PDGFRA | na | 33905 | -0.477 | -0.0264 | No |
| 89 | PIK3R5 | na | 34383 | -0.611 | 0.0100 | No |
Table: GSEA details [plain text format]

  

Fig 2: KEGG\_PROSTATE\_CANCER      
 Blue-Pink O' Gram in the Space of the Analyzed GeneSet

  

Fig 3: KEGG\_PROSTATE\_CANCER: Random ES distribution      
 Gene set null distribution of ES for **KEGG\_PROSTATE\_CANCER**
